# Transient lymphadenopathy during intravenous immunoglobulin (IVIg) in a patient with pemphigus vulgaris

**DOI:** 10.1002/ccr3.4763

**Published:** 2021-09-15

**Authors:** Delila Pouldar Foulad, Michael Rigas, Fawad Piracha, Meeta Gupta, Maisie Nguyen, Sergei A. Grando, Shehla Memon

**Affiliations:** ^1^ Department of Dermatology University of California Irvine California USA; ^2^ KabaFusion Cerritos California USA

**Keywords:** intravenous immunoglobulin, lymphadenopathy, pemphigus vulgaris

## Abstract

This is the first case of transient cervical lymphadenopathy as an adverse event during IVIg infusion. IVIg plays a vital role in the treatment of many dermatological conditions and identification of adverse events can facilitate patient counseling.

## Graphical abstract

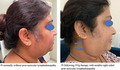

## 1 INTRODUCTION

Pemphigus is a family of autoimmune blistering skin dermatoses caused by IgG autoantibodies targeting keratinocyte adhesion molecules, leading to loss of cell‐to‐cell adhesion and an intraepithelial split.^1^ Pemphigus vulgaris (PV) is associated with autoantibodies against desmoglein 1 (Dsg1) and desmoglein 3 (Dsg3), and manifested by flaccid bulla on mucosa and sometimes also the skin. Pemphigus foliaceus (PF) is associated with autoantibodies directed to Dsg1, and patients present clinically with disseminated crusted erosions and sometimes with fragile blisters on the skin.^1^

Intravenous immunoglobulin (IVIg) has become a core component of pemphigus treatment by lowering circulating serum autoantibodies.^1^ In contrast to systemic corticosteroids and immunosuppressive agents, IVIg has a more favorable adverse event profile and is not associated with increased infection risk. A retrospective analysis showed that of 123 pemphigus patients, 100% achieved disease remission with the following multidrug protocol: a loading dose of prednisone, prolonged administration of IVIg, an immunosuppressive cytotoxic drug, and mitochondrion protecting drugs.^2^ Reported adverse events of IVIg in that study included mild to severe intensity headache, nausea/vomiting, fever/chills, fatigue, changes in blood pressure, and cutaneous symptoms, such as pruritus, erythema, and urticaria.

Herein, we report a case of a female with PV who experienced transient lymphadenopathy during IVIg infusion.

## 2 CASE REPORT

A 46‐year‐old female with a four‐year history of PV presented to the dermatology clinic with new oral erosions and a tongue fissure consistent with active disease. Her diagnosis of PV was supported by direct immunofluorescence (DIF) of a skin biopsy, indirect immunofluorescence (IIF) of serum, and ELISA for anti‐Dsg1 and anti‐Dsg3 antibodies. The multidrug protocol^2^ was initiated with a prednisone taper, IVIg (Octagam® 2 g/kg per month divided into 5 consecutive daily infusions), mycophenolate mofetil 1000 mg BID, doxycycline 100 mg BID, niacinamide 500 mg TID, calcium supplementation, and a multivitamin. During her course, the patient was transitioned off from mycophenolate mofetil to rituximab 700 mg IV weekly as she was refractory to treatment.

**FIGURE 1:**
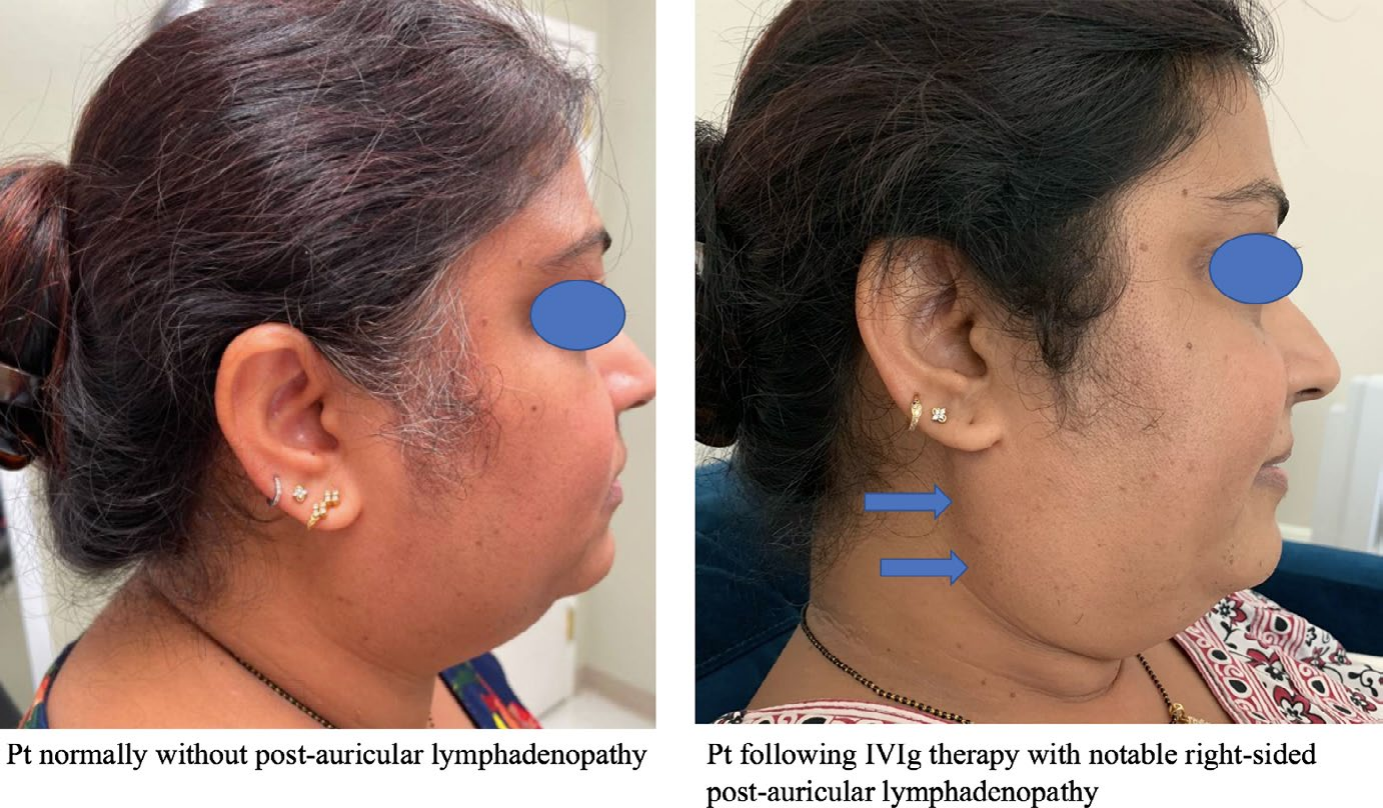
Transient right‐postauricular lymphadenopathy during IVIg infusion with resolution following cessation of the infusion and diphenhydramine 25 mg IM

During the fourth day of her IVIg infusion, the patient was noted to have a triangular‐shaped, nontender nodule behind her right ear after receiving 12 grams of IVIg at an infusion rate of 75 ml/h. Vital signs remained stable and the patient denied any preceding illness. Examination demonstrated a 3 × 4 cm soft nodule within the right posterior triangle of the neck, consistent with postauricular lymphadenopathy (Figure 1). The IVIg infusion was stopped, diphenhydramine IM 25 mg was administered, and the nodule resolved within 30 min. When the infusion was restarted at a slower rate of 50 ml/h, the lymphadenopathy reappeared within two minutes, and once again resolved within minutes of stopping the infusion and administering the antihistamine. IVIg infusion was once again restarted at a rate of 75 ml/h, and the patient completed the treatment uneventfully. The lymphadenopathy did not recur with subsequent IVIg treatments. The Naranjo algorithm or Adverse Drug Reaction Probability Scale indicates a “probable” causal relationship between the patient's lymphadenopathy and the IVIg administration.

## 3 DISCUSSION

This is the first reported case of transient lymphadenopathy arising during an IVIg infusion for the treatment of pemphigus. A review of the *FDA Adverse Events Reporting System (FAERS)* public dashboard in October 2020 revealed a rare incidence of lymphadenopathy (5/930 (0.54%)) following IVIg treatment in all reported patients, which did not stratify for patients receiving IVIg for blistering dermatoses.^3^ A retrospective review by *Gürcan* et al.^4^ found that of 103 patients with PV and 15 patients with PF, only one patient developed a lymph node enlargement. All nodes were noted after completion of IVIg and lasted 3–4 days (mean: 3.2 days). Computed tomography (CT) of the neck were within normal limits and the symptoms did not recur after IVIg infusions were discontinued.^4^ Interestingly, in our case lymphadenopathy resolved with antihistamines within minutes of stopping the infusion. The transient nature of the lymphadenopathy is a limitation that did make imaging or physician evaluation possible.

Thus, our case serves to present the first case of transient cervical lymphadenopathy as an adverse event during IVIg infusion.

## CONFLICT OF INTEREST

None declared.

## AUTHOR CONTRIBUTIONS

Category 1 Conception and design of study: Michael Rigas, PharmD, Fawad Piracha PharmD, IgCP, Meeta Gupta, PharmD, IgCP, Maisie Nguyen, PharmD, AAHIVP, Shehla Memon, MD. Acquisition of data: Shehla Memon, MD. Analysis and/or interpretation of data: Shehla Memon, MD. Category 2. Drafting the manuscript: Delila Pouldar Foulad MD. Revising the manuscript critically for important intellectual content: Delila Pouldar Foulad MD, Fawad Piracha PharmD, IgCP, Sergei A. Grando MD, PhD, Dsc, Shehla Memon, MD. Category 3. Approval of the version of the manuscript to be published (the names of all authors must be listed): Delila Pouldar Foulad MD, Michael Rigas, PharmD, Fawad Piracha PharmD, IgCP, Meeta Gupta, PharmD, IgCP, Maisie Nguyen, PharmD, AAHIVP, Sergei A. Grando MD, PhD, Dsc, Shehla Memon, MD.

## Data Availability

Data sharing is not applicable to this article as no datasets were generated or analyzed during the current study.

## References

[1] Kershenovich R, Hodak E, Mimouni D. Diagnosis and classification of pemphigus and bullous pemphigoid. Autoimmun Rev. 2014;13(4‐5):477‐481. 10.1016/j.autrev.2014.01.011 24424192

[2] Grando SA. Retrospective analysis of a single‐center clinical experience toward development of curative treatment of 123 pemphigus patients with a long‐term follow‐up: efficacy and safety of the multidrug protocol combining intravenous immunoglobulin with the cytotoxic immunosuppressor and mitochondrion‐protecting drugs. Int J Dermatol. 2019;58(1):114‐125. 10.1111/ijd.14143 30047585

[3] Food and Drug Administration,USA . “FDA Adverse Events Reporting System (FAERS) Public Database–Octagam Human Immunoglobulin”. FDA Adverse Events Reporting System (FAERS) Public Database, 20 Sept. 2020, fis.fda.gov/sense/app/d10be6bb‐494e‐4cd2‐82e4‐0135608ddc13/sheet/45beeb74‐30ab‐46be‐8267‐5756582633b4/state/analysis

[4] Gürcan HM, Ahmed AR. Frequency of adverse events associated with intravenous immunoglobulin therapy in patients with pemphigus or pemphigoid. Ann Pharmacother. 2007;41(10):1604‐1610. 10.1345/aph.1K198 17785614

